# Prevalence, Antibiotic Resistance Pattern, and Molecular Characteristics of *Staphylococcus epidermidis* Isolated From Milk of Pure Breeds of Dairy Cattle With Subclinical Mastitis

**DOI:** 10.1155/jotm/8893420

**Published:** 2025-12-03

**Authors:** Farhad Badshah, Naseem Rafiq, Mustafa Kamal, Mourad Ben Said, Shehryar Khan, Irfan Khattak, Naimat Ullah Khan, Aljawharah Fahad Alabbad, Tahir Usman

**Affiliations:** ^1^College of Veterinary Sciences and Animal Husbandry, Abdul Wali Khan University Mardan, Mardan 23200, Pakistan; ^2^Department of Zoology, Abdul Wali Khan University Mardan, Mardan 23200, Pakistan; ^3^State Key Laboratory of Animal Biotech Breeding, Institute of Animal Science, Chinese Academy of Agricultural Science, Beijing 100193, China; ^4^Guangdong Laboratory of Lingnan Modern Agriculture, Key Laboratory of Livestock and Poultry Multi-Omics of MARA, Agricultural Genomics Institute at Shenzhen, Shenzhen Branch, Chinese Academy of Agricultural Sciences, Shenzhen 518000, China; ^5^Department of Basic Sciences, Higher Institute of Biotechnology of Sidi Thabet, University of Manouba, Manouba 2010, Tunisia; ^6^Laboratory of Microbiology, National School of Veterinary Medicine of Sidi Thabet, University of Manouba, Manouba 2010, Tunisia; ^7^Department of Biotechnology, Abdul Wali Khan University Mardan, Mardan 23200, Pakistan; ^8^Botany and Microbiology Department, College of Science, King Saud University, Riyadh 11451, Saudi Arabia

**Keywords:** antibiotic resistance, bovine subclinical mastitis, multidrug resistance, Pakistani dairy cattle, resistant genes, *Staphylococcus epidermidis*

## Abstract

Bovine mastitis, a widespread disease in dairy cattle characterized by udder inflammation triggered primarily by pathogenic micro-organisms, poses a considerable challenge to the dairy industry. *Staphylococcus epidermidis (S. epidermidis)* stands out as a significant etiological factor in the incidence of bovine subclinical mastitis (SCM), further exacerbated by the diminishing efficacy of antibiotics due to the increase in antibiotic-resistant strains. This study sets out to comprehensively investigate the landscape of *S. epidermidis* in dairy cattle afflicted with SCM. We examined the prevalence of *S. epidermidis*, assessed its antibiotic resistance patterns, and probed for the presence of antibiotic-resistant genes (*mecA*, *tetK*, and *ermC*) within *S. epidermidis* strains isolated from 305 milk samples across four distinct dairy cattle breeds: Holstein Friesian, Red Sindhi, Sahiwal, and Cholistani. Among the sampled cows, 56.39% (172 out of 305) were found to have SCM. Within this group, *S. epidermidis* was identified in 27.90% (48 out of 172) of the cases. Our breed-specific analysis revealed significant disparities, with Red Sindhi cows displaying the highest prevalence at 75%, followed by Holstein Friesian at 45.45%, and significantly lower levels in Sahiwal (5.19%) and Cholistani (3.44%) breeds. To assess the efficacy of antibiotics, we conducted sensitivity testing using nine commonly prescribed antibiotics. Alarmingly, 18 out of the 48 isolates (37.5%) exhibited multidrug resistance (MDR). Our antibiogram results underscored a high resistance of *S. epidermidis* isolates, particularly against cefoxitin (56.25%) and penicillin (43.75%), while demonstrating remarkable susceptibility to amikacin (2.08%), clindamycin (0%), ciprofloxacin (0%), and chloramphenicol (0%). Furthermore, we employed PCR to ascertain the presence of resistant genes in all *S. epidermidis* isolates. *mecA* was detected in 38 isolates (79.16%), while *tetK* was identified in 33 isolates (68.75%). Notably, the study did not detect the presence of the *ermC* gene. Our investigation highlights the efficacy of chloramphenicol, clindamycin, and ciprofloxacin against *S. epidermidis*. However, the prevalence of multidrug-resistant strains calls for careful antibiotic use in veterinary practices. Further research is needed to examine geographic and farm-specific factors affecting *S. epidermidis* prevalence, and genetic techniques like multilocus sequence typing should be employed to study clonal spread and horizontal gene transfer. Routine antimicrobial sensitivity assessments and continuous monitoring of medication use are essential to develop sustainable strategies against antibiotic resistance in the dairy industry.

## 1. Introduction

The term “mastitis” has its roots in the Greek words “mastos” (referring to the mammary gland) and “itis” (indicating inflammation), making it the medical terminology for the inflammation of the mammary gland. A multitude of pathogens have been identified as culprits responsible for causing mastitis [1]. When these pathogens invade the mammary gland and teat canal, they undergo proliferation and produce toxins that result in structural damage, subsequently leading to a decline in milk production and quality [2, 3]. Mastitis can manifest in two distinct forms: clinical or subclinical, contingent on the presence of observable symptoms. Clinical mastitis (CM) is characterized by sudden udder swelling, redness, diminished appetite, high fever, and noticeable changes in the udder and milk composition. Conversely, subclinical mastitis (SCM) lacks apparent symptoms in the udder or milk but contributes to a reduction in milk production and an increase in somatic cell counts (SCCs) [4]. SCM can substantially diminish the milk yield and, in addition, negatively impact the composition and nutritional value of the milk, rendering it less suitable for human consumption [5]. SCM surpasses CM in terms of prevalence and poses a substantial challenge in dairy farm settings, potentially serving as a reservoir for udder infection-causing micro-organisms in animals [6]. Several factors contribute to the risk of SCM, encompassing host-related parameters such as breed, age, lactation stage, teat injuries, and genetic resistance, along with pathogen-related factors such as indiscriminate treatment, the quantity of infectious micro-organisms, and virulence factors [5]. In a global context, antibiotic therapy is widely employed to mitigate intramammary infections in dairy cattle. However, its efficacy against mastitis pathogens, particularly *Staphylococcus* species, is limited due to the emergence of antibiotic resistance in these bacteria [7]. Antibiotics are extensively used in the treatment and prevention of diseases in dairy cattle [8]. Nonetheless, it is important to acknowledge that no antibiotic can completely eradicate all target bacteria within the intricate microbial ecosystems of these animals [9]. The misuse of antibiotics has resulted in multidrug resistance (MDR), a primary factor contributing to the low cure rate of staphylococcal pathogens [10]. Research has underscored that mastitis milk functions as a significant reservoir for MDR strains, with an increasing prevalence of coagulase-negative, methicillin-resistant staphylococci, particularly *S. epidermidis*, found in the mastitic milk of cows. This underscores the critical importance of monitoring and regulating antibiotic use in mastitis management to curb the development of MDR strains [11, 12]. Notably, antibiotics from different classes, including tetracyclines, aminoglycosides, cephalosporins, fluoroquinolones, macrolides, and penicillin, have exhibited limited efficacy against *S. epidermidis* strains. This highlights the immediate need to explore alternative approaches for the prevention and treatment of mastitis infections caused by these resistant strains [13].

Although *Staphylococcus aureus* and *Streptococcus agalactiae* remain the classical major mastitis pathogens in dairy cattle, *S*. *epidermidis* has recently emerged as one of the most prevalent coagulase-negative staphylococci (CNS) isolated from SCM cases. Its significance stems from several unique features: the ability to form biofilms, which enhance its persistence in the mammary gland; frequent MDR complicating treatment; and its role as a reservoir of antimicrobial resistance genes that may be horizontally transferred to other mastitis pathogens [12–14]. These characteristics justify prioritizing *S. epidermidis* alongside the traditional major pathogens in research and control strategies. Epidemiologically, *S. epidermidis* is frequently isolated from mastitic milk, especially in subclinical cases, contributing to economic losses due to the reduced milk yield and quality [6, 11]. Despite not always ranking “at the top” compared to *S. aureus* or *S. agalactiae*, increasing evidence highlights its growing importance in bovine udder health. This study specifically targeted three antibiotic resistance genes with high clinical and epidemiological relevance in staphylococci. The *mecA* gene encodes Penicillin-Binding Protein 2a (PBP2a), conferring methicillin and other β-lactam resistance, serving as a marker for methicillin-resistant staphylococci [15, 16]. The *tetK* gene encodes a tetracycline efflux pump, mediating tetracycline resistance, a class of antibiotics widely used in livestock [13]. The *ermC* gene encodes an rRNA methylase responsible for macrolide resistance, such as erythromycin [17]. These genes represent critical mechanisms influencing therapeutic management of mastitis and justify their inclusion in this study.

Therefore, the present study is designed to assess the prevalence and antibiotic resistance patterns of *S. epidermidis* and evaluate the presence of various antibiotic-resistant genes (*mecA*, *tetK*, and *ermC*) in milk samples obtained from selected pure breeds of dairy cattle experiencing SCM. This includes investigating whether breed-related genetic and physiological differences influence *S. epidermidis* prevalence and resistance patterns, with the aim of providing insights for targeted management and breeding strategies.

## 2. Materials and Methods

### 2.1. Study Design and Sample Collection

In this cross-sectional study, we collected a total of 305 milk samples at different stages of lactation from four distinct purebred lactating dairy cow breeds, including Holstein Frisian, Sahiwal, Cholistani, and Red Sindhi. These samples were sourced from multiple government dairy farms in Pakistan representing diverse management practices across the region. Each milk sample was meticulously collected in prelabeled sterile bottles that featured breed and animal identification numbers. Approximately 10 mL of milk was aseptically collected from each lactating quarter during morning milking. Prior to collection, teats were cleaned with sterile water, dried with disposable paper towels, and disinfected with 70% ethanol. The first few streams of milk were discarded, and midstream milk samples were collected into prelabeled sterile screw-capped tubes (Oxoid, UK) marked with animal ID and breed. Immediately after collection, the samples were placed in an insulated ice box at 4°C and transported to the Laboratory of Genetics and Animal Breeding at Abdul Wali Khan University Mardan. Until further bacteriological analysis, all samples were stored at 4°C and processed within 24 h of collection.

The sampling procedure was conducted in strict accordance with institutional and international guidelines for the care and use of animals. Ethical approval for this study was obtained from the Institutional Animal Ethics Committee of Abdul Wali Khan University Mardan (Approval No. Dir/A&A/AAWKUM/2022/9396). Written informed consent was also obtained from farm owners prior to sample collection.

### 2.2. Diagnosis of SCM

SCM was diagnosed by analyzing the milk samples for SCC by microscopy. Initially, approximately 0.1 mL of milk was carefully placed on clean glass slides and allowed to air-dry at room temperature for 15–30 min to ensure complete evaporation of moisture. Once dried, the samples were defatted by immersing them in xylene for 2–3 min to remove fat globules. After defatting, the slides were fixed with 95% ethanol for 5 min, which preserved the cellular structure. Following fixation, the samples were stained with 10% Giemsa stain for 5–10 min, allowing for effective visualization of cells. The slides were then rinsed gently with tap water to remove excess stain and air-dried. The SCC was ascertained by examining the prepared slides under a microscope using a 100X oil immersion lens. The number of somatic cells was counted in multiple fields of view to ensure accuracy, and the results were noted as per guidelines reported by Zajác et al. [18]. Dairy cows were divided into three categories based on their SCC in their milk: Group 1 for low SCC (below 200,000 cells/mL), Group 2 for moderate SCC (between 200,000 and 500,000 cells/mL), and Group 3 for high SCC (over 500,000 cells/mL) in their milk samples.

### 2.3. Bacterial Culturing and Biochemical Identification

The milk samples were cultured on 7% Mannitol salt agar (MSA) obtained from Oxoid Ltd., Hampshire, England. The incubation took place for a duration of 16–18 h at 37°C under aerobic conditions. After this incubation period, the plates were examined for the growth of *S. epidermidis*. Small white pinpointed colonies that appeared on the incubated plates were indicative of the presence of *S. epidermidis*. To ensure the purity of the isolates, they were subcultured on fresh MSA. Final confirmation was achieved through biochemical tests, including a positive catalase test and a negative coagulase test.

### 2.4. Extraction of Genomic DNA and *S. epidermidis* Molecular Identification

The DNA of all *S. epidermidis* isolates was extracted using a standardized protocol [19]. Molecular identification of all *S. epidermidis* isolates was carried out through PCR, utilizing the species-specific *rdr* gene, following the protocol as previously documented by Venugopal et al. [20]. Detailed information about the primer sequences can be found in Table 1.

### 2.5. Antibiotic Susceptibility Testing

Antibiotic susceptibility testing of all *S. epidermidis* isolates was conducted using the disc diffusion method in adherence to Clinical and Laboratory Standards Institute (CLSI) guidelines 2013. A panel of eight antibiotics, including penicillin (10 μg), clindamycin (10 μg), erythromycin (15 μg), cefoxitin (30 μg), amikacin (30 μg), tetracycline (30 μg), ciprofloxacin (15 μg), and chloramphenicol (30 μg), was employed. Mueller–Hinton agar (MHA) obtained from Oxoid Ltd., Hampshire, England, was meticulously prepared following the manufacturer's instructions. The MHA plates were then inoculated with *S. epidermidis* using a sterile cotton swab. Subsequently, the plates were incubated for 24 h at 37°C. The zones of inhibition around each antibiotic disc were measured, and interpretations were made in accordance with CLSI 2013 guidelines reported by Dos Santoset al. [23].

### 2.6. Detection of Antibiotic-Resistant Gene

The study employed a well-established protocol for the detection of antibiotic-resistant genes (*tetK*, *mecA*, and *ermC*) using PCR with primers provided by Bio-Rad Inc., USA, as detailed in Table 1. A reaction mixture of 20 μL for *tetK* and *mecA* was prepared, comprising 10 μL of master mix (BioBasic Inc., Canada), 6 μL of PCR-grade water, 2 μL of the DNA sample, and 1 μL each of the forward and reverse primers (Bio-Rad Inc., USA). The thermal cycling protocol involved an initial denaturation at 95°C for 10 min, followed by 30 cycles consisting of denaturation at 95°C for 30 s, annealing at 56.60°C for 30 s (*tetK*) and 60.20°C for 30 s (*mecA*), extension at 72°C for 30 s, and a final elongation at 72°C for 10 min. Detection of the *ermC*-resistant gene followed established protocols from prior studies [15, 17, 24]. The PCR products were analyzed by gel electrophoresis on a 2% agarose gel (Bio-Rad Inc., USA) containing 3 μL of ethidium bromide (Bio-Rad Inc., USA).

### 2.7. Statistical Analysis

Data collected from government dairy farms were entered into Excel spreadsheets and analyzed using SPSS Statistics Version 26. To investigate the association between the prevalence of *S. epidermidis* and cattle breeds, a chi-square test of independence was applied. This test was also used to assess the differences in SCC group distributions and antibiotic resistance patterns. Statistical significance was set at *p* < 0.05. Where applicable, post hoc pairwise comparisons with Bonferroni correction were performed to identify specific differences between breeds. Odds ratios with 95% confidence intervals were calculated to quantify the strength of associations, with the Cholistani breed as the reference group. This analytical framework follows the methodology outlined by Farabi et al. [25].

## 3. Results

### 3.1. Detection of SCM by SCC

Milk samples from 305 dairy cows were classified into three groups based on their SCC values. Group 1 consisted of 133 cows with SCC < 200,000 cells/mL, representing healthy animals. Group 2 included 63 cows with SCC between 200,000 and 500,000 cells/mL, indicative of mild SCM. Group 3 comprised 109 cows with SCC > 500,000 cells/mL, reflecting severe SCM (Table 2). Overall, 172 cows were diagnosed with SCM, yielding a prevalence of 56.39%. These SCC thresholds comply with internationally recognized standards where an SCC ≥ 200,000 cells/mL is considered the diagnostic cutoff for SCM, correlating with udder inflammation and infection risk. Monitoring SCC, thus, provides a reliable indicator for early detection and herd health management.

### 3.2. Isolation and Confirmation of *S. epidermidis*

**Figure 1:**
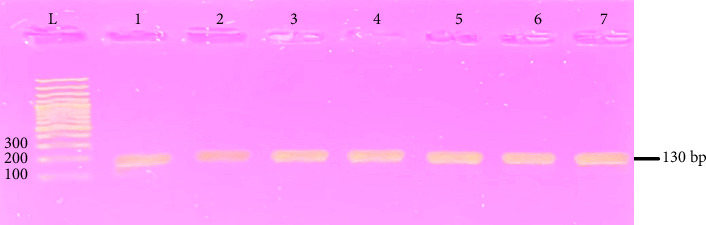
Molecular identification of *S. epidermidis*. L; DNA ladder; 1–7: positive samples.

Out of the 172 samples that tested positive for SCM, all were subjected to culturing and subculturing on MSA growth media. Among these samples, 48 (27.90%) exhibited distinct pinpointed white colonies characteristic of *S. epidermidis*. These 48 isolates underwent additional biochemical confirmation through gram staining, coagulase testing, and catalase testing. Following successful biochemical confirmation, molecular identification was carried out using PCR, targeting the species-specific *rdr* gene, which produced the expected 130 bp amplicon.  Figure 1 shows the representative PCR results, where Lanes 1–7 correspond to positive isolates of S. epidermidis producing clear bands at 130 bp, alongside a DNA ladder (L). These results confirm the molecular identity of the isolates as *S. epidermidis.*

### 3.3. Prevalence of *S. epidermidis* in Different Breeds of SCM Dairy Cattle

*S. epidermidis* prevalence varied significantly among the investigated breeds diagnosed with SCM. The highest prevalence was observed in the Red Sindhi breed, with 75% (33/44) of SCM cows testing positive. This was followed by Holstein Friesian at 45.45% (10/22). In contrast, the Sahiwal and Cholistani breeds exhibited considerably lower prevalence rates of 5.19% (4/77) and 3.39% (1/29), respectively (Table 3). Statistical analysis revealed the differences in prevalence between breeds were highly significant (*p* < 0.0001), indicating a strong breed-associated variation in susceptibility or colonization by *S. epidermidis*.

### 3.4. Antibiotic Susceptibility Test Result

A total of 48 *S. epidermidis* isolates were tested against nine commonly used antibiotics. Among these, 18 isolates were identified as multidrug-resistant, exhibiting resistance to three or more antibiotic classes. The highest resistance rate was observed for cefoxitin, with 56.25% of isolates resistant, followed by penicillin at 43.75%. Conversely, isolates demonstrated no resistance to clindamycin, ciprofloxacin, and chloramphenicol, each showing a 0% resistance rate. Amikacin resistance was low at 2.08% (Table 4).

### 3.5. Detection of Antibiotic-Resistant Genes

**Figure 2:**
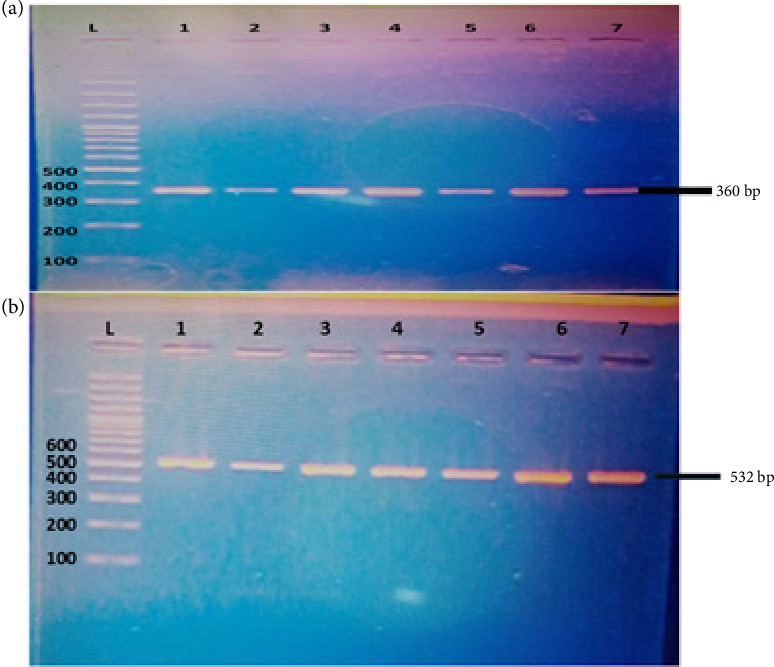
Detection of *tetK* (a) and *mecA* (b) antibiotic-resistant genes. L; DNA ladder; 1–7: positive samples.

A PCR analysis was conducted on all 48 *S. epidermidis* isolates to detect the presence of antibiotic-resistant genes (*mecA, tetK,* and *ermC*). The results showed that 38 samples contained the *mecA* gene, indicating a prevalence rate of 79.16%, while 33 samples contained the *tetK* gene, resulting in a prevalence rate of 68.75% (Figures 2(a) and 2(b)). Remarkably, the *ermC* gene was not detected in this study.

## 4. Discussion

Bovine mastitis, characterized by inflammation of the udder, remains a significant concern in the dairy industry, exacting a substantial economic toll by diminishing milk production and quality [26]. SCM, a precursor to clinical symptoms, emerged as the focus of our study, and its prevalence was estimated at approximately 56.39%. Notably, this prevalence surpasses what has been reported in earlier research. For instance, Contreras et al. [27] reported lower prevalence rates in dairy goats, while Ndahetuye et al. [28] identified higher prevalence at the cow level but lower prevalence at the quarter level. In contrast, Ali et al. [29] recorded a lower prevalence in the Punjab province.

The elevated prevalence observed in our study may be attributed to various factors within the dairy farming landscape. Poor milking practices, inadequate hygiene protocols, delayed treatment, and the failure to segregate infected cows from healthy ones all contribute to this higher prevalence of SCM. Understanding these contributing factors is crucial for devising effective strategies to reduce the incidence of this condition in dairy cattle. Proper milking practices, stringent hygiene protocols, timely treatment, and isolation of infected cows are key areas that require attention to mitigate the economic and animal welfare implications of bovine mastitis. Moreover, this research sheds light on the urgency of adopting best practices within the dairy industry to safeguard udder health and, by extension, milk production and quality. Our study unveiled a significant prevalence of *S. epidermidis*, reaching 27.90%, among dairy cows with SCM. This finding aligns with the prevalence reported in prior research, which documented prevalence rates of 25% and 26.8%, respectively, in mastitis cases [30, 31]. A study by Argudín et al. [14] in pigs in Belgium also revealed a similar prevalence of 28% in mastitis cases. In contrast, our observed prevalence exceeded the rates reported by Kudinha et al. [32], Junaidu et al. [33], Hosseinzadeh et al. [34], and Sumathi et al. [35], whose reported prevalence rates were 7.4%, 10.9%, 10.2%, and 16%, respectively. Likewise, Delgado et al. [36], Las Heras et al. [37], and Contreras et al. [38], observed higher *S. epidermidis* infection rates in mastitis cases, with prevalence figures of 74%, 40%, and 66.7%, respectively. Waller et al. [39] reported a higher prevalence of 40% in both SCM and CM. These variations in prevalence rates underline the multifaceted nature of factors influencing the prevalence of *S. epidermidis* in dairy cows. Regional differences, farm management practices, and variations in diagnostic methods can all contribute to these disparities.

The higher prevalence of *S. epidermidis* in cases of SCM, as demonstrated in our study, calls for an exploration of the underlying factors. Notably, the transmission of this pathogen may be linked to poor milking practices and a lack of stringent hygiene protocols, where contaminated hands come into contact with milking machines. Comparing our findings with existing literature, reported by Barkema et al. [40] and Leitner et al. [41], it becomes evident that *S. epidermidis* prevalence in mastitis cases can exhibit substantial variability across different geographic regions and farm management practices. This underscores the complex interplay of factors influencing the prevalence of this pathogen in dairy cattle. Future research endeavors should delve deeper into the intricate facets of cow health, farm management, and milking hygiene to enhance our understanding of and mitigation strategies for the prevalence of *S. epidermidis* in mastitis cases. This study did not employ multilocus sequence typing (MLST) to assess the clonal or horizontal spread of *S. epidermidis* isolates, limiting our understanding of genetic diversity and transmission dynamics. Future research should incorporate MLST to address these epidemiological questions. Such insights will be instrumental in developing more effective preventive measures and management strategies within the dairy industry.

Our current investigation revealed varying prevalence rates of *S. epidermidis* across different cattle breeds. Red Sindhi and Holstein Friesian breeds exhibited the highest prevalence, standing at 75% and 45.45%, respectively, while Sahiwal and Cholistani breeds displayed the lowest prevalence rates, at 5.19% and 3.44%, respectively. The variation in *S. epidermidis* prevalence among different cattle breeds could be influenced by genetic, physiological, and management factors. The higher prevalence in the mentioned breeds may be linked to increased genetic susceptibility and environmental conditions. This outcome aligns with earlier research, underscoring the influence of breed as a significant factor in mastitis prevalence. For example, our findings on the prevalence of *S. epidermidis* are consistent with those of Guo et al. [42], who reported a notable distribution of *S. epidermidis* among pathogens responsible for CM on large Chinese dairy farms. Their study also highlights that *S. epidermidis* is a predominant cause of mastitis in high-producing dairy breeds, which supports our observation of higher prevalence in Holstein Friesians compared to indigenous breeds such as Sahiwal and Cholistani. The consistency between our findings and Guo et al.'s study also suggested that breed susceptibility, potentially linked to genetic and physiological differences, plays a critical role in *S. epidermidis* prevalence in dairy herds.

In our study, antimicrobial susceptibility testing disclosed that 18 out of 48 (37.5%) *S. epidermidis* isolates were classified as MDR. Notably, *S. epidermidis* displayed considerable resistance to cefoxitin at 56.25% and penicillin at 43.75%. In contrast, minimal resistance was observed for clindamycin, ciprofloxacin, and chloramphenicol (0%), along with amikacin at 2.08%. This resistance pattern echoes findings reported by Onni et al. [43], Chabi and Mumtaz [13], and Xu et al. [16], who similarly documented high resistance of *S. epidermidis* to penicillin. Further studies by Xu et al. [16], Du et al. [21], and Chajęcka-Wierzchowska et al. [22] have consistently reported notable resistance of *S. epidermidis* to Penicillin particularly in isolates harboring the *mecA* gene, which confers resistance to methicillin and other β-lactam antibiotics. The *mecA* gene was detected in 79.16% of our isolates, indicating a strong association between this gene and resistance to these antibiotics. The observed high resistance to penicillin and cefoxitin may be attributed to their overuse over extended periods, while the selective use of chloramphenicol, clindamycin, and ciprofloxacin may account for their lower resistance. The prevalence of resistance to penicillin and cefoxitin may, in part, be linked to the inappropriate prescription and usage of these antibiotics. These findings emphasize the importance of prudent antibiotic use and the need for ongoing efforts to combat antibiotic resistance within veterinary practices.

Phenotypic variation between *S. epidermidis* strains plays a crucial role in resistance patterns. Our findings are consistent with previous reports that emphasize the importance of phenotypic typing to understand the behavior of antibiotic-resistant strains, especially in dairy cattle environments [13]. This insight reinforces the need for prudent use of antibiotics in veterinary practices to curb the spread of MDR strains. The current study identified the *mecA* and *tetK* resistant genes as highly prevalent in *S. epidermidis*, with prevalence rates of 79.16% and 68.75%, respectively. Remarkably, no instances of the *ermC* gene were detected in any of the isolates. This aligns with findings reported by Chabi and Mumtaz [13], and Asante et al. [44], while Tang et al. [45] documented a lower prevalence of the *ermC* and *tetK* genes. The detection of the *mecA* gene is significant in the treatment of mastitis in cows, as it signifies resistance to antibiotics from the methicillin group. On the other hand, the *tetK* gene plays a role in conferring resistance to tetracycline antibiotics, while the *ermC* gene operates by modifying rRNA methylation or employing an efflux system to resist erythromycin antibiotics. However, the erythromycin resistance observed in *S. epidermidis* in our study did not correlate with the presence of the *ermC* gene, implying the involvement of other factors in antibiotic resistance. Additionally, the observed discrepancy between the high prevalence of the *tetK* gene (68.75%) and the low phenotypic resistance to tetracycline (6.25%) may be explained by regulatory mechanisms affecting gene expression or other genetic and environmental factors. Investigating the conditions under which *tetK* is expressed functionally is a critical area for further exploration. Furthermore, our study suggests that resistant genes represent only one facet of the complex landscape of antibiotic resistance, highlighting the need for a comprehensive understanding of the underlying mechanisms in *S. epidermidis*.

## 5. Conclusion

This study reveals breed-related variations in *S. epidermidis* prevalence among dairy cattle with SCM, with Red Sindhi cattle showing the highest susceptibility. The observed MDR, notably against Cefoxitin and Penicillin, underscores the need for prudent antibiotic use in veterinary practices. The presence of *mecA* and *tetK* resistant genes raises concerns about antibiotic treatment. This complexity emphasizes the importance of multifaceted management approaches, including breed-specific prevention strategies, proper milking practices and stringent hygiene protocols, to combat *S. epidermidis* prevalence and MDR within the dairy industry. Further studies incorporating geographic variability, longitudinal sampling, and genetic analysis including MLST are recommended to clarify the prevalence, transmission dynamics, and genetic diversity of *S. epidermidis*. Additionally, exploring the functional expression of resistant genes will help better understand their role in antimicrobial resistance and the development of MDR.

## Figures and Tables

**Table 1:** Primers used in the molecular identification of *S. epidermidis* and the detection of antibiotic- resistant genes.

Target gene	Primer sequence (5′-3′)	Amplicon size (bp)	Reference
*rdr*	F: AAGAGCGTGGAGAAAAGTATCAAG	130	[21]
	R: TCGATACCATCAAAAAGTTGG		

*tetK*	F: AAAATCGATGGTAAAGGTTGGC	360	[13]
	R: AGTTCTGCAGTACCGGATTTGC		

*mecA*	F: GTAGCGACAATAGGTAATAGT	532	[13]
	R: GTAGTGACAATAAACCTCCTA		

*ermC*	F: AATCGTCAATTCCTGCATGT	299	[22]
	R: TAATCGTGGAATACGGGTTTG		

**Table 2:** Distribution of SCC groups across dairy cattle breeds.

SCC groups	Red Sindhi	Holstein Friesian	Sahiwal	Cholistani	Total (*n*)	% Red Sindhi	% Holstein Friesian	% Sahiwal	% Cholistani
Group 1 (Healthy; SCC < 200,000 cells/mL)	60	18	23	32	133	44.8	31.0	23.0	53.3
Group 2 (Mild SCM; SCC 200,000–500,000 cells/mL)	29	8	17	9	63	21.6	13.8	17.0	15.0
Group 3 (Severe SCM; SCC > 500,000 cells/mL)	15	14	60	20	109	11.2	24.1	60.0	33.3
Total (*n*)	104	40	100	61	305				

*Note:* Chi-square test of independence showed a statistically significant association between cattle breed and SCC group distribution (*χ*^2^ = 45.87, df = 6, *p* < 0.001); percentages are calculated as the proportion within each breed for every SCC group; SCM = Subclinical mastitis.

**Table 3:** Prevalence of *S. epidermidis* in dairy cattle with subclinical mastitis by breed.

Breed	Total examined	Cattle with SCM	*S. epidermidis* positive	Prevalence rate (%)	95% C.I.	Odds ratio (vs. Cholistani)	95% C.I. (OR)	Post hoc *p* value
Red Sindhi	104	44	33	75.00	60.7–86.4	36.6	4.41–304.03	< 0.0001
Holstein Friesian	40	22	10	45.45	24.4–67.8	17.3	1.83–163.57	0.003
Sahiwal	100	77	4	5.19	1.4–12.8	1.57	0.16–15.14	0.84
Cholistani	61	29	1	3.39	0.09–17.6	—	—	—
Total	305	172	48	27.91	21.6–34.9	—	—	—

*Note:* Chi-square test statistic = 80.23; *p* < 0.0001; odds ratios compare each breed to Cholistani (reference lowest prevalence); post hoc tests (Bonferroni-corrected): significant differences Red Sindhi vs others (*p* < 0.0001); Holstein Friesian vs Cholistani (*p*=0.003); differences for Sahiwal not significant (*p* > 0.05); 95% C.I. for prevalence from binomial exact method (Clopper–Pearson).

**Table 4:** Antibiotic susceptibility pattern of *S. epidermidis* isolates (*n* = 48).

Antibiotic	Quantity (μg)	Resistant (*n*)	Resistant (%)	Intermediate (*n*)	Intermediate (%)	Sensitive (*n*)	Sensitive (%)
Penicillin (P)	10	27	56.25	0	0	21	43.75
Clindamycin (CD)	10	24	50.00	24	50.00	0	0
Erythromycin (E)	15	34	70.83	11	22.92	3	6.25
Cefoxitin (FOX)	30	21	43.75	0	0	27	56.25
Amikacin (AK)	30	2	4.17	1	2.08	45	93.75
Tetracycline (TE)	30	0	0	3	6.25	45	93.75
Ciprofloxacin (CIP)	5	8	16.67	0	0	40	83.33
Chloramphenicol (C)	30	0	0	0	0	48	100

## Data Availability

The data that support the findings of this study are available from the corresponding author upon reasonable request.
